# Interspecies RNA Interactome of Pathogen and Host in a Heritable Defensive Strategy

**DOI:** 10.3389/fmicb.2021.649858

**Published:** 2021-07-21

**Authors:** Marcela Legüe, Blanca Aguila, Andrea Calixto

**Affiliations:** ^1^Centro Interdisciplinario de Neurociencia de Valparaíso, Instituto de Neurociencia, Facultad de Ciencias, Universidad de Valparaíso, Valparaiso, Chile; ^2^Programa de Doctorado en Microbiología, Universidad de Chile, Santiago, Chile

**Keywords:** small RNAs, *C. elegans*, *P. aeruginosa* PAO1, interspecies communication, host behavioral defenses, RNA–RNA interaction, dual-RNA-seq transcriptomics, pathogen-induced diapause

## Abstract

Communication with bacteria deeply impacts the life history traits of their hosts. Through specific molecules and metabolites, bacteria can promote short- and long-term phenotypic and behavioral changes in the nematode *Caenorhabditis elegans*. The chronic exposure of *C. elegans* to pathogens promotes the adaptive behavior in the host’s progeny called pathogen-induced diapause formation (PIDF). PIDF is a pathogen avoidance strategy induced in the second generation of animals infected and can be recalled transgenerationally. This behavior requires the RNA interference machinery and specific nematode and bacteria small RNAs (sRNAs). In this work, we assume that RNAs from both species co-exist and can interact with each other. Under this principle, we explore the potential interspecies RNA interactions during PIDF-triggering conditions, using transcriptomic data from the holobiont. We study two transcriptomics datasets: first, the dual sRNA expression of *Pseudomonas aeruginosa* PAO1 and *C. elegans* in a transgenerational paradigm for six generations and second, the simultaneous expression of sRNAs and mRNA in intergenerational PIDF. We focus on those bacterial sRNAs that are systematically overexpressed in the intestines of animals compared with sRNAs expressed in host-naïve bacteria. We selected diverse *in silico* methods that represent putative mechanisms of RNA-mediated interspecies interaction. These interactions are as follows: heterologous perfect and incomplete pairing between bacterial RNA and host mRNA; sRNAs of similar sequence expressed in both species that could mimic each other; and known or predicted eukaryotic motifs present in bacterial transcripts. We conclude that a broad spectrum of tools can be applied for the identification of potential sRNA and mRNA targets of the interspecies RNA interaction that can be subsequently tested experimentally.

## Introduction

Bacteria and animals are adapted to living together, and their interaction impacts the physiology of both entities (Rosenberg and Zilber-Rosenberg, 2013; Moënne-Loccoz et al., 2015). Many small molecules and metabolites are known to be mediators of this communication (Cleary et al., 2017). The emerging relevance of RNA molecules in the bacteria–host interplay is based on the discovery of specific bacterial RNAs that directly induce phenotypic changes in hosts. For instance, *Escherichia coli* non-coding RNAs (ncRNAs) OxyS and DsrA affect chemotaxis and longevity in *Caenorhabditis elegans* by downregulating *che-2* (Liu et al., 2012). *Salmonella enterica* microRNA-like RNA fragment Sal-1 targets host-inducible nitric oxide synthase (Zhao et al., 2017); *Pseudomonas aeruginosa* PA14 methionine-transfer RNAs (tRNA) fragment from outer membrane vesicles (OMVs) induces IL-8 secretion in human epithelial cells (Koeppen et al., 2016); ncRNA p11 induces intergenerational learned avoidance in *C. elegans* by targeting *maco-1* (Kaletsky et al., 2020); and *P. aeruginosa* PAO1 RsmY triggers transgenerational diapause (Legüe et al., 2021).

The aforementioned works support the idea that RNAs from both species can be transferred between animal tissues and be co-expressed spatially and temporally. This transcriptomic layer of regulation of the bacteria–host *holobiont* (Celluzzi and Masotti, 2016; Westermann et al., 2016; Rosenberg and Zilber-Rosenberg, 2018) is called *holo-transcriptome* (Palominos et al., 2017; Legüe and Calixto, 2019) and implies a tight communication directly at the RNA expression level. Indirect evidence of RNA exchange came from the fact that RNA is selectively secreted in bacterial extracellular vesicles and exosomes, which could be transferred between organisms (Ghosal et al., 2015; Sjöström et al., 2015; Celluzzi and Masotti, 2016; Koeppen et al., 2016; Liu et al., 2016; Westermann et al., 2016; Lefebvre and Lécuyer, 2017). Despite the advances in understanding the small RNA (sRNA)-mediated bacteria–host interaction, the broad spectrum of mechanisms by which RNAs from both species could interact has not been fully explored.

The search for inter-kingdom RNA interactions represents a conundrum. On one hand, the regulation of intraspecies RNA–RNA interaction is complex and diverse. On the other hand, mechanisms in bacteria and eukaryotes differ, making the process of finding commonalities between them a challenge. Until now, the search for interspecies RNA communication has relied on an oversimplification of the possibilities, using RNA sequence similarity as the only parameter (Nguyen et al., 2018). Here, we aim to systematize the possible mechanisms of interspecies RNA interaction and evaluate *in silico* the applicability of different bioinformatics tools for each proposed mechanism. To accomplish this, we take advantage of a behavioral paradigm in *C. elegans* called pathogen-induced diapause formation (PIDF) (Palominos et al., 2017; Gabaldón et al., 2020), which involves sRNAs from both bacteria and hosts. PIDF is a strategy of defense by which animals enter diapause to effectively avoid feeding on pathogens. For PIDF to take place, RsmY from *P. aeruginosa* (Legüe et al., 2021) and *mir-243* from *C. elegans* (Gabaldón et al., 2020) are required. In this paradigm, animals are in contact with pathogens for two generations and are re-introduced to pathogens after two generations in non-pathogenic bacteria. The datasets we use include intergenerational and transgenerational sRNA and polyA+ RNA-seq transcriptomics. This allows the comparison of generations of animals from the same cohort feeding on pathogens and non-pathogens. Additionally, this design permits the discrimination between dynamic changes in sRNA expression from the constitutive expression that are mostly due to intestinal life. Among RNA species that emerged as players of interspecies interaction are the tRNAs, which we show are implicated in the process of PIDF.

In this work, we explore interspecies RNA–RNA interactions based on the mechanisms described for bacteria and Eukarya independently. For each putative RNA interaction mechanism, we test and select the most appropriate existing bioinformatics tool. Finally, from the predicted relevant players, we show that ELPC-3, a tRNA elongation factor, plays a role in PIDF. These analyses open new insights and interesting lines of research in interspecies communication.

## Materials and Methods

### Datasets Used

For downstream interaction prediction, we use the following datasets: (1) *C. elegans* sRNA transcriptomics deposited in the NCBI under BioProject no. PRJNA659467 (Gabaldón et al., 2020); (2) *P. aeruginosa* PAO1 sRNA transcriptomics deposited in the NCBI under BioProject no. PRJNA708299 (Legüe et al., 2021); (3) polyA+ transcriptomics to obtain downregulated mRNAs and lncRNAs during bacteria–nematode interaction, from Supplementary Table 2 (Gabaldón et al., 2020) available at https://mbio.asm.org/content/mbio/11/5/e01950-20/DC2/embed/inline-supplementary-material-2.xlsx; and (4) normalized sRNA expression from *P. aeruginosa* PAO1 and *C. elegans* (Legüe et al., 2021).

Small RNA libraries were based on size selection of fragments shorter than 200 nt. Based on these criteria, we use the broad term of sRNA for transcripts shorter than 200 nt, and we refer to specific biotypes when relevant.

The data obtained from the *C. elegans* polyA+ RNA transcription was pre-processed using Trimmomatic v. 0.36 (Bolger et al., 2014). Reads with a quality (Phred score) less than 35 and a length less than 36 were removed. The reads were aligned using TopHat (Trapnell et al., 2009) and quantified with HTSeq (Anders et al., 2015). Differentially expressed genes (DEGs) were determined using EdgeR (Robinson et al., 2010) and DeSeq (Anders and Huber, 2010). DEG cutoff was set with a *p*-adjusted value < 0.01.

The sRNA data from *P. aeruginosa* and *C. elegans* sRNAs (Gabaldón et al., 2020; Legüe et al., 2021) was previously processed as follows unless explained otherwise:

#### Small RNA Data Pre-processing and Quality Control

Quality visualization was made with FastQC^1^. Trimming was performed with Cutadapt (Martin, 2011), using Diagenode-recommended parameters for CATS Library Preparation Kits available at https://www.diagenode.com/en/documents/diagenode-trimming-tools-for-cats-rnaseq.

#### Mapping

For each sample, reads were mapped to the *E. coli* OP50 genome assembly ASM435501v1 or *P. aeruginosa* PAO1, assembly ASM676v1, available at NCBI^2^ as appropriate, using Bowtie2 version 2.2.6 (Langmead and Salzberg, 2012) with one allowed mismatch and seed set to 17 base pairs. As a result, a bam file was produced for each sample.

#### Detection of Previously Unannotated Transcripts

Units of expression were defined as transcriptional peaks (TP) following the methodology described in Gabaldón et al. (2020). Briefly, the TPs were generated by merging all the bam files and selecting the coordinates of the expression peaks (more than 10 per base). For the subsequent analysis, the transcripts with moderate or high expression (10 or more reads per nucleotide) were kept. Finally, a comparison of the TP obtained with annotations reported in databases was made and classified according to their genomic context.

### Matching Genomic Sequences Between Species

To identify nearly perfect complementary transcripts between *C. elegans* and *P. aeruginosa*, we first needed to distinguish those reads belonging to each species. To this end, we considered that *bona fide* bacterial transcripts need to be expressed in naïve *P. aeruginosa*. We aligned reads from the sRNA transcriptome of naïve bacteria against the *C. elegans* genome with Bowtie2 (Langmead and Salzberg, 2012), allowing one mismatch and setting the seed to 17 bp. Next, to evaluate the expression of these transcripts in intestinal bacteria, we mapped reads from the holobiont bacteria–nematode against *C. elegans* (WBcel235) and *P. aeruginosa* PAO1 genome with the same parameters. Finally, we counted the naïve bacterial reads that mapped *C. elegans* features with HTSeq-count. Reads from the bacteria–nematode holobiont that were indistinguishable were counted against the features that previously matched with *P. aeruginosa*-naïve reads.

### Matching Transcriptional Expression in the Holobiont

Transcriptional peaks from *P. aeruginosa* PAO1 in dataset from Legüe et al. (2021) were compared with *C. elegans* TPs in dataset from Gabaldón et al. (2020) by Reciprocal Best Hit (RBH) analysis using BLAST+ (Altschul et al., 1990; Camacho et al., 2009). The rationale is that if two genes in different genomes are the best hit of each other in the other genome, they are orthologs candidates (Moreno-Hagelsieb and Latimer, 2008). We performed a reciprocal blast using nucleotides and with a discontiguous megablast task, which is optimized for interspecies comparison. Our dataset contained short sequences ranging from 18 to 200 nt. Therefore, to avoid spurious matches, we set the *e*-value < 0.01 for considering a hit.

### Bacterial RNAs Systematically Overexpressed During Bacteria–Host Interaction

*Pseudomonas aeruginosa* sRNAs overexpressed in the intestines of the F1, F2, F5, and F6 nematode generations compared to naïve bacteria were operationally defined as *induced sRNAs*. We defined overexpression as a log2 fold change 1.5 and *p*_*adj*_ < 0.01. *P. aeruginosa* sRNAs whose expression level did not change during exposure to the animal intestine compared to the naïve condition were called *constitutive*. We selected those with log2 fold change less than 0.5 and *p*_*adj*_ value > 0.85. As input for selecting *induced and constitutive sRNAs* in *P. aeruginosa*, we used the differential expression data reported in Legüe et al. (2021). This sRNA annotation was based on TPs that allowed the identification of RNA fragments based on their genomic coordinates (Gabaldón et al., 2020; Legüe et al., 2021).

### Bacterial microRNA-Like sRNAs Targeting Host mRNAs

We use the rationale that bacterial transcripts with a size similar to microRNAs (miRNAs) could act over host mRNAs using miRNA-induced post-transcriptional inhibition. We defined putative bacterial miRNA-like genes as those transcripts or fragments with size between 17 and 28 nt, filtered from Legüe et al. (2021). We selected as potential targets of miRNA-like RNAs the host-downregulated mRNAs reported in Gabaldón et al. (2020). The putative interspecies interactions between miRNA-sized-induced sRNAs from bacteria and host-downregulated mRNAs were assessed with IntaRNA 2.0 (Mann et al., 2017). We set the temperature parameter to 20°C and seed length as default.

### Eukaryotic Regulatory Motifs Contained in Bacterial Transcripts

The functional eukaryotic motifs we focused on are listed in Supplementary File 2. For the identification of these motifs in the sequences of bacterial sRNA transcripts, the online tool RegRNA 2.0 (Chang et al., 2013) was used. This tool identifies regulatory RNA motifs and elements in mRNA sequences by integrating information on regulatory elements from data sources and analytical approaches (Chang et al., 2013). The *induced* and *constitutive* sRNAs mentioned above were used as input sequences. The Fisher’s exact test was applied to discriminate the statistical significance of motifs in *induced* and *constitutive* sRNAs.

### Enrichment Analysis

Enrichment analysis for tissue, phenotype, and gene ontology for *C. elegans* genes that are putative targets of the induced sRNAs from bacteria was performed using the enrichment tool from www.wormbase.org database (Angeles-Albores et al., 2016, 2018).

### Cross-Kingdom RNA Network Construction

For constructing this network, we assumed that sRNAs coming from bacteria and nematodes are co-localized and co-expressed. The network was constructed using as nodes those *P. aeruginosa* RNAs that emerged as *induced*, *constitutive*, *microRNA-sized*, and *common TPs*, from the predictions of this work. Additionally, *C. elegans* transcripts that qualified as targets in any of the aforementioned predictions were also used as nodes. Attributes considered for nodes were differential expression, their organism, and their biotype, and for bacteria, whether the sRNAs were *induced* or *constitutive* and microRNA-sized. Edge types considered the inhibitory or activating potential of interaction and their putative interaction mechanism. Edges were based on all the cross-kingdom interactions predicted in this paper. Statistical analysis and graph visualization were performed in Gephi 0.9.2. To understand the general structure of the network, statistical analysis was executed with the built-in tools in Gephi 0.9.2, to calculate the following metrics: average degree, graph density, modularity, Eigenvector centrality, average path length, and average cluster coefficient. For graph visualization, a Force Atlas layout was set. Adjusted repulsion strength was adjusted to 500, maintaining the other parameters as default. Node sizes were ranked by betweenness centrality, and color partitions were made by biotype.

### Data Availability

Raw data were deposited in the NCBI under BioProject no. PRJNA659467. All the scripts used in these analyses are availableat Bitbucket: https://bitbucket.org/srnainterspeciesinteraction/workspace/projects/ISIS_RNA.

### *C. elegans* and Bacterial Growth

Wild-type and mutant *C. elegans* strains were grown at 20°C as previously described (Brenner, 1974). All nematode strains were grown on *E. coli* OP50 before pathogen exposure. *P. aeruginosa* PAO1 (ATCC 15692) was used for infection protocols. Bacteria were grown overnight on Luria-Bertani (LB) plates at 37°C from glycerol stocks. The next morning, a large amount of the bacterial lawn is inoculated in LB broth and grown for 6 h at 250 rpm and at 37°C. Three milliliters of the resulting bacterial culture is seeded onto 90-mm NGM plates and allowed to dry for 36 h before worms are placed on them.

### *C. elegans* Strains

We used the following strains of the *Caenorhabditis* Genetics Center (CGC): wild type (N2), VC1937 (*elpc-2*), and VC463 (*rsp-2*).

### *C. elegans* Growth in Pathogenic Bacteria

Five L4 (P0) wild-type worms or mutants previously maintained in *E. coli* OP50 were picked and transferred to a 90-mm-diameter plate seeded with 3 ml of *P. aeruginosa* PAO1 or *E. coli* OP50 control bacteria. In all cases, the bacterial lawn covered the plate. After 8 days, the total number of worms and dauer larvae were quantified. The number of bacteria seeded allowed animals to be well fed for the length of the experiment. If worms starved, the experiment was discarded. Each assay was performed in three independent experiments (technical replicates) generating a biological replica. A total of three biological replicates were considered for each analysis.

### Quantification of Population and Dauer Larvae

#### Dauer Formation on Pathogens

Entire worm populations on each plate were collected in 1 ml of M9. This initial stock was diluted 1:10 in M9. To count the total population of worms under a Nikon SMZ745 stereomicroscope, 10 μl of this 1:10 dilution was used. To quantify the number of dauers in each population, the initial stock was diluted 1:10 in a 1% SDS solution and maintained in constant agitation for 20 min (Cassada and Russell, 1975). To count the number of total animals and dauers, 10 μl of this last dilution was placed in a glass slide under the stereomicroscope. Each condition was scored three times (triplicates of each technical replica), and dauers were plotted as a percentage of the total populations of animals.

## Results

### Matching sRNA Expressed Sequences From Bacteria With the Genome of *C. elegans*

**FIGURE 1:**
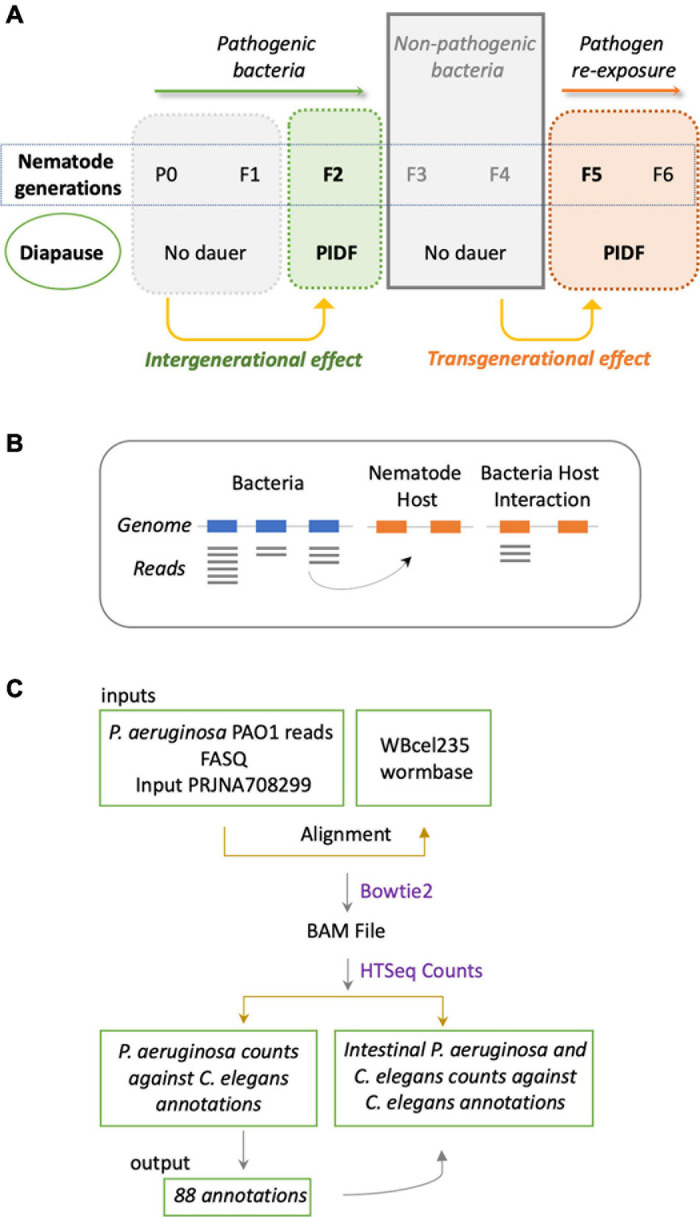
**(A)** Schematic representation of the inter- and transgenerational paradigm of pathogen-induced diapause formation (PIDF). Animals chronically feeding on pathogenic bacteria such as *P. aeruginosa* PAO1 form dauers in the second generation of exposure to the bacteria. Re-exposure to pathogens after two generations on non-pathogenic bacteria triggers the immediate formation of the dauer larvae. **(B)** Representation of the putative mechanism of interaction based on matching sRNA sequences from bacteria with genomic sequences of *C. elegans*. **(C)** Methodological flow to address the mechanism proposed in panel **(B)**.

The sustained interaction between *P. aeruginosa* PAO1 and *C. elegans* triggers PIDF, a protective response to infection in host progenies (Palominos et al., 2017; Figure 1A). PIDF requires the RNA interference machinery of the host and is maintained transgenerationally. We hypothesize that cross-kingdom RNA interactions in the holobiont underlie this behavior. From many possible mechanisms of RNA–RNA interaction, we first examined the common sequences that could act as a *cis*-encoded regulatory element in bacteria (Waters and Storz, 2009; Storz et al., 2011) or as small interfering RNAs in the nematode host. In this scenario, interspecies sequences would interact with nearly perfect complementarity. To prove this, we compared public genomic data and transcriptomic data generated in a previous work (Legüe et al., 2021) from bacteria grown on standard LB broth (naïve bacteria) and for six generations in the transgenerational paradigm shown in Figure 1A. We searched for sRNAs from naïve *P. aeruginosa* PAO1 that matched regions of the *C. elegans* genome (Figure 1B). We aligned the *P. aeruginosa* PAO1 reads against the *C. elegans* genome WBcel235 with Bowtie2 (Langmead and Salzberg, 2012). Accordingly, from the cross-mapped reads between the two species, we selected those with annotations in the nematode (Figure 1C). We found that naïve *P. aeruginosa* reads map to 88 *C. elegans* annotations (Supplementary Table 1). Half of these features matched genes that can be expressed either as coding or non-coding isoforms (Supplementary Figure 1A). Matching non-coding sRNAs include ncRNA, Piwi-interacting RNAs (piRNA), tRNA, and one pre-miRNA. The *C. elegans* gene with the most abundant matching reads from naïve *P. aeruginosa* is the ribosomal RNA *rrn-3.1* (Supplementary Figure 1B). Because the sequence identity potentially allows the interaction between the two transcripts, we speculate that if this mechanism occurs, it should reflect in expression changes upon intestinal interaction between the two species. Therefore, we evaluated the expression of matching *P. aeruginosa*–*C. elegans* sequences (genome level) in the condition of bacteria colonizing the nematode intestine (transcription level) taking data from dual RNA-sequencing experiments mentioned before. We focused on matching transcripts highly expressed in naïve bacteria (more than 10 TMM, Table 1) and explored their expression change in the first and second intestinal generations: While *rrn-3.1* matching sequences in *P. aeruginosa* increased dramatically in read number in the intestinal conditions, all other genes that were highly expressed in naïve bacteria dropped their expression. Based on that, we conclude that genomic matches are not insightful enough to assert physiological relevance.

**TABLE 1:** Non-coding *C. elegans* genes matching bacterial reads with high expression in host-naïve and intestinal conditions.

***C. elegans* annotation**	**Biotype**	**Bacterial Read Count (TPMs)**		
		**Host-Naïve**	**F1**	**F2**
*rrn-3.1*	rRNA	74997.5	164489.2	119781.3
*21ur-6043*	piRNA	144471.0	1958.7	3130.2
*mir-235*	pre_miRNA	46295.1	327.6	175.9
K07C10.5	ncRNA	292653.5	164.3	659.5
M163.t1	tRNA	13745.4	160.8	88.8
C06E4.17	ncRNA	50543.3	135.2	988.4
C02F4.11		2221.5	129.7	83.8
*21ur-3672*	piRNA	19341.1	95.8	0.0
Y43F8A.8	ncRNA	7011.9	84.1	158.2
C43H6.11		7908.7	59.0	98.6
W05B2.13		2937.3	40.0	152.8
K05G3.t1	tRNA	5368.3	34.5	0.0
W03G11.8	ncRNA	4838.9	34.1	17.3

### Matching Transcriptional Expression in the Holobiont

**FIGURE 2:**
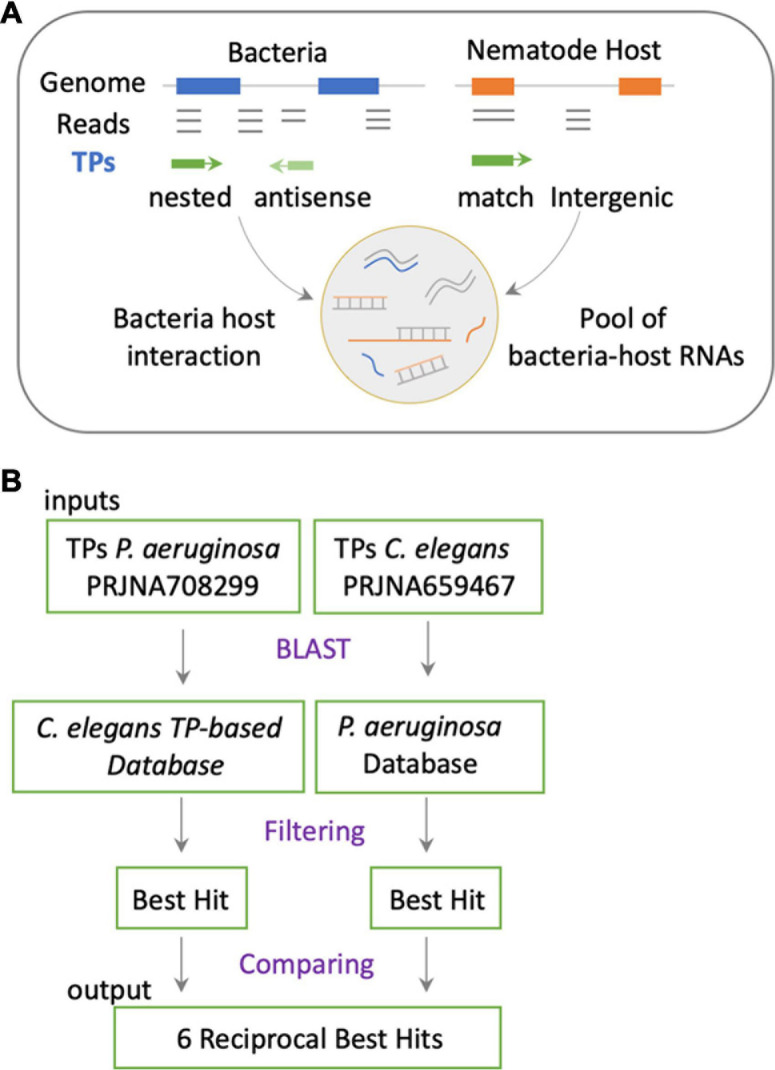
**(A)** Representation of the putative mechanism of interaction based on transcripts expressed in holobiont and that have the potential of sequence matching. This approach considers actual transcriptomic data of both species in a synchronous paradigm. **(B)** Methodological flow to address the mechanism proposed in panel **(A)**.

The cross-mapping approach (Figures 1B,C) has two main conceptual gaps: (1) the lack of information on how reads are distributed along feature coordinates; and (2) the uncertainty of whether reads are simultaneously co-expressed in both organisms to make possible their interaction. To address these shortcomings, we used synchronous dual RNA-sequencing data generated by us of bacteria and nematodes under PIDF-inducing conditions, both intergenerational (Gabaldón et al., 2020) and transgenerational (Legüe et al., 2021). In these datasets, the annotation of transcripts was based on TPs, which consider pervasive transcription (Lybecker et al., 2014) and add genomic context information (Gabaldón et al., 2020). We classify the TPs as matching annotations, nested, or overlapped in genomic features or novel fragments situated in intergenic regions. The relevance of this approach is that it uncovers functional sRNA fragments previously unannotated or intragenic sense-encoded transcripts (Huang et al., 2010). We hypothesize that co-expressed sequences in both species could mimic each other or form heterologous RNA–RNA duplexes (Figure 2A), changing the RNA homeostasis by affecting regulatory loops or RNA sponge occupation (Azam and Vanderpool, 2015). To define interspecies common sequences, we looked for perfectly matching sequences between expressed sequences, by RBH analysis with the BLAST+ tool (Camacho et al., 2009). Figure 2B shows the workflow, where we used the TPs expressed in *P. aeruginosa* (454) and *C. elegans* (7,964) during any generation of their interaction (Legüe et al., 2021). Four transcripts mutually hit each other on the same strand and one in the opposite strand (Table 2). These hits are nested in proteins with similarly described functions, as seen in Table 2, suggesting that they could be functional orthologs.

**TABLE 2:** Reciprocal best hits of TPs expressed in *P. aeruginosa* PAO1 and *C. elegans* simultaneously.

***P. aeruginosa***		***C. elegans***			***P. aeruginosa–C. elegans* interaction**		
**Gene name**	**Protein name**	**Gene name**	**Protein name**	**Tissue**	**% Identity**	**mismatches**	***E*-value**
rhlE	ATP-dependent RNA helicase	*laf-1*	RNA strand annealing activity; RNA-dependent ATPase	P granule/germline	80	8	6.54E-05
Lpd	Dihydrolipoamide dehydrogenases	*dld-1*	Dihydrolipoyl dehydrogenase activity and flavin adenine dinucleotide binding activity	Germline precursor cell	78	9	0.003
SucC	Succinyl-CoA syntetase	*suca-1*	Succinate-CoA ligase ADP-forming subunit beta	Anterior gonad arm	82	9	1.49E-07
GlgP	Glycogen phosphorylase	*pygl-1*	Glycogen phosphorylase and pyridoxal phosphate binding activity	Germline	81	9	8.11E-07
hscA	Heat shock protein	*hsp-1(s)/hsp-2(as)*	Heat shock protein/pseudogene	Germline and intestine	80	9	6.35E-06

*s, sense; as, antisense.*

### A Core of Bacterial RNAs Is Systematically Overexpressed During Bacteria–Host Interaction

**FIGURE 3:**
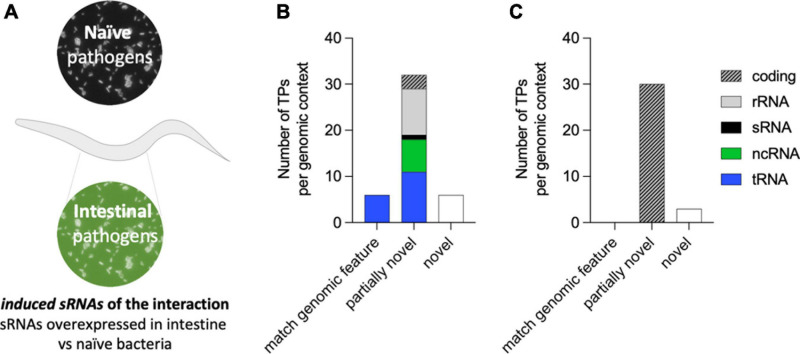
**(A)** Diagram of the concept of bacterial-induced small RNAs (sRNAs). Induced sRNAs are those bacterial transcripts showing upregulation in all intestinal conditions (F1, F2, F5, and F6) compared to their expression in naïve bacteria. **(B,C)** RNA biotypes and genomic contexts of the induced bacterial sRNA **(B)** and constitutively expressed sRNAs **(C)** from *P. aeruginosa*. The genomic context category of matching genomic feature refers to transcripts that fit the size and genomic coordinate of an existing feature, and the category partially novel refers to transcripts that are either nested or overlapping a previously annotated genomic feature. The category novel refers to a transcript matching an intergenic region of the genome.

The colonization of the *C. elegans* intestine by *P. aeruginosa* for two continued generations is a requisite for PIDF to take place and highlights the bidirectional molecular interaction between microbe and host (Palominos et al., 2017). This interaction also leaves a memory that allows the defensive strategy to be established immediately upon reencounter (F5 in paradigm of Figure 1A). We reasoned that pathogen sRNAs with a role in triggering PIDF and the subsequent transgenerational memory are overexpressed in the intestines of generations F1, F2, F5, and F6 compared to host-naïve bacteria (Figure 3A), herein *induced RNAs* (log2FC > 0.5 and *p*_*adj*_ < 0.01). Forty-four genes were upregulated in intestinal bacteria in the four generations compared to the naïve condition (Supplementary Table 2). According to our rationale, these bacterial genes would generate molecular interactions with host genes that are conducive to behavioral changes. On the other hand, we hypothesized that genes constitutively expressed in both intestinal and naïve conditions (log2FC < 0.5 and *p*_*adj*_ > 0.85) are unresponsive to the interaction with the nematode, herein *constitutive sRNAs*. Thirty-three genes were constitutively expressed in naïve and intestinal conditions (Supplementary Table 3). Interestingly, induced RNAs were predominantly nested in non-coding transcripts such as tRNAs and rRNAs (Figure 3B), while constitutive sRNAs were mostly nested in coding transcripts (Figure 3C). At the moment, we do not know the implication of this finding, but it may highlight the importance of transcription in non-coding regions in the adaptation to new environments. Throughout the following text, the *induced RNAs* and their comparison with constitutively expressed RNAs serve as a source for predictive interaction analysis.

### Exploring the miRNA-Like Mechanisms of Bacterial sRNAs Targeting Host mRNA

**FIGURE 4:**
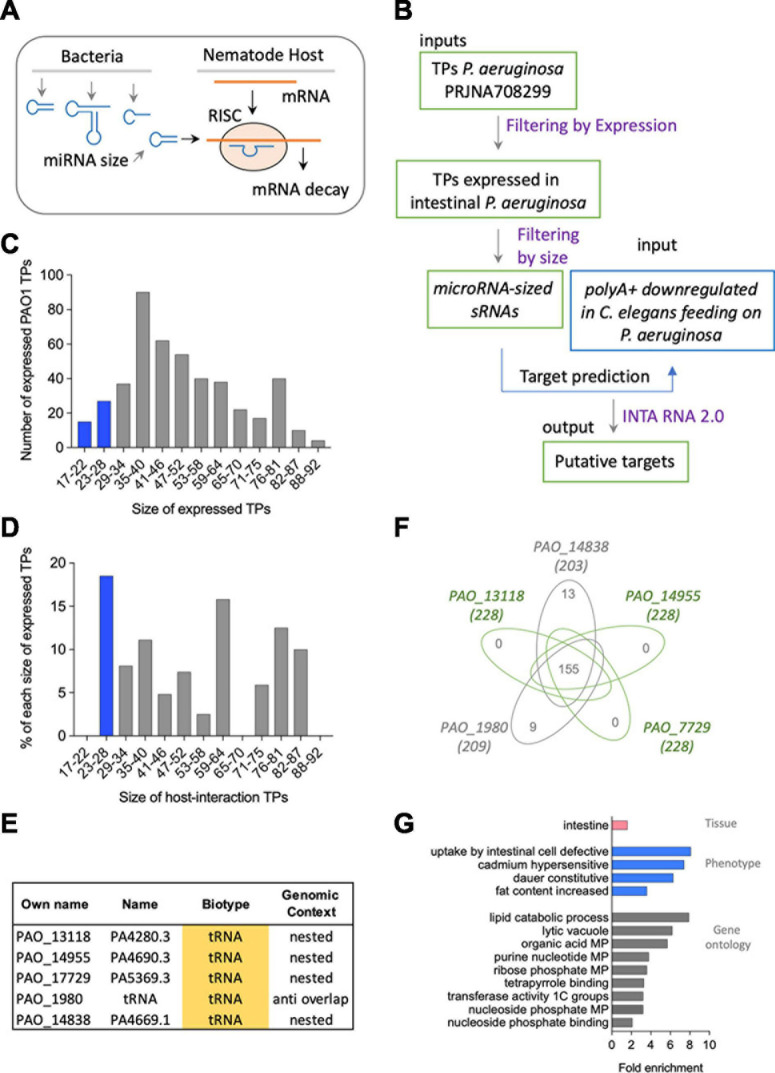
**(A)** Representation of the putative mechanism of interaction based on *miRNA-like* mechanisms of bacterial sRNAs targeting host mRNA. **(B)** Methodological flow to address the mechanism proposed in panel **(A)**. **(C)** Size and number of expressed transcriptional peaks (TPs) in *P. aeruginosa* PAO1. **(D)** Size and number of induced RNA TPs in *P. aeruginosa* PAO1. **(E)** Biotype and genomic context of *microRNA-sized*-induced TPs in *P. aeruginosa* PAO1. **(F)** Venn diagram of mRNAs targeted by the five miRNA-like-induced sRNAs. **(G)** Tissue (pink), phenotype (blue), and gene ontology (gray) enrichment of mRNAs targeted by the five miRNA-like-induced sRNAs.

Bacterial sRNAs can regulate host mRNAs using the RNAi machinery in a similar way to an endogenous microRNA (Cardin and Borchert, 2017; Zhao et al., 2017; Figure 4A). This mechanism is analogous to post-transcriptional inhibition by bacterial *trans*-encoded base-pairing RNAs, which act via limited base pairing (Waters and Storz, 2009; Storz et al., 2011). We aimed to find feasible miRNA-like interactions between the *induced* sRNAs from *P. aeruginosa* and *C. elegans* mRNAs. To that end, we first selected from all TPs from bacteria expressed upon interaction with the nematode (Legüe et al., 2021) those with a length between 17 and 28 nucleotides, hereby *microRNA-sized* (Figure 4B). From the 456 bacterial genes expressed in either F1, F2, F5, or F6 nematode generations (Supplementary Table 4), we found 44 *microRNA-sized* transcripts (Figure 4C). Among those, five were *induced sRNAs*, all of which were either nested or antisense to tRNAs (Figures 4D,E). Conversely, two of the constitutively expressed transcripts was *microRNA-sized*, one of them nested in a protein-coding gene and one novel (Supplementary Table 5).

Based on the expectation that the regulation of a target by a miRNA is repressive, we used as putative targets 313 downregulated polyA+ *C. elegans*-coding genes upon PIDF (Gabaldón et al., 2020) and their isoforms. We predicted miRNA-like interactions using the IntaRNA 2.0 tool (Mann et al., 2017), using a cutoff of −8 (Trotta, 2014) for the minimum free energy (MFE) of interaction. Two hundred thirty-six genes and 1,096 isoforms surpass this cutoff (Supplementary File 1). The complete interaction data and selected datasets are shown in Supplementary File 1. We speculate that the more times a polyA+ is predicted to be a target of a bacterial RNA, the probability of being a *bona fide* target increases. One hundred fifty-five mRNAs were targeted by the five *microRNA-sized*-induced sRNAs (Figure 4F and Supplementary Table 6). The 155 targets had three putative sites for the binding of each *microRNA-sized* sequence. The candidate target genes (155) were subject to tissue, phenotype, and gene ontology enrichment analysis (Angeles-Albores et al., 2016, 2018). These genes show intestinal expression in *C. elegans* with a phenotypical enrichment in intestinal uptake (Figure 4G). We analyzed the interactions for all *microRNA-sized* sRNAs expressed in any of the intestinal generations (Supplementary Tables 4, 7). Supplementary Figure 2 shows that the five *microRNA-sized* sRNAs and the expressed sRNAs in any intestinal generation share most predicted targets. This suggests that the expression and not necessarily the overexpression of a bacterial sRNA can impact the global pool of mRNAs in the host.

### Eukaryotic Regulatory Motifs Contained in Bacterial-Induced sRNAs

**FIGURE 5:**
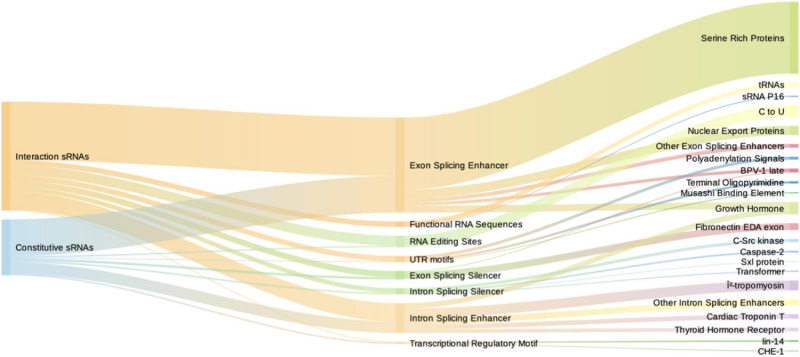
Sankey diagram depicting the number of motifs of induced sRNAs (red) and constitutive sRNAs (blue) in the first column, motif type in the second, and specific targets in the third column. Intron and exon splicing regulatory motifs are similarly represented among induced and constitutive sRNAs. Functional RNA sequences (comprising tRNAs and sRNA P16) are exclusive from induced sRNAs.

Numerous functional RNA motifs in eukaryotes control gene expression. These motifs are defined by specific sequences or secondary structures that bind or are targeted by transcriptional or translational regulators, such as transcription factors (Morris, 2011), splicing enhancers or inhibitors, RNA editing sites, and other functional sequences (Mattick and Makunin, 2006). To the extent of our knowledge, the presence of the aforementioned motifs in bacterial RNAs has not been explored in an interaction paradigm such as PIDF. Therefore, we aimed to identify bacterial-induced sRNAs motifs that could potentially bind eukaryotic gene expression regulators. We tested a wide variety of motif types potentially present in *induced RNAs* by using RegRNA 2.0 (Figure 5; Chang et al., 2013). As a control, we performed the same analysis with the constitutive sRNAs whose expression is not modified during interaction.

We found that induced and constitutive RNAs did not differ in the total number of eukaryotic-regulatory motifs (Supplementary File 2), both displaying a high number of them. The common motifs shared by induced and constitutive sRNAs (total number of 146 and 77, respectively) were varied. By far, the most frequent motifs were *splicing regulators* (114/146 and 68/77). In both *induced* and *constitutive* sRNAs, the exon splicing enhancers (ESE) predominate with 52 and 62%, respectively, followed by intron splicing enhancer (ISE) (16.9 and 19.4%, respectively) motifs. Interestingly, the most frequent ESE motifs were members of the serine-rich protein family, such as *srp40* (27/114–23.7% of splicing regulatory motifs). s*rp40* and *srp55* are homologous to *C. elegans* SR protein RSP-2, which is involved in larval development. This suggests that commonly expressed bacterial sRNAs should carry different splicing regulatory motifs. However, in our paradigm, their presence is not increased with the interaction with hosts. However, the type of motifs was different between groups, being the *functional regulatory sequences* and *RNA editing sites* significantly overrepresented in the *induced sRNAs* (Fisher’s exact test, *p* = 0.038 and *p* = 0.04, respectively). The *functional regulatory sequences* corresponded mainly to tRNA motifs (6/7) and p16, also termed RgsA, involved in quorum sensing and regulated by GacS/GacA system (Brencic et al., 2009). This could suggest a role of tRNAs and their modifications in interspecies interaction.

### Integrative Analysis of the Cross-Kingdom RNA Interactome Network

**FIGURE 6:**
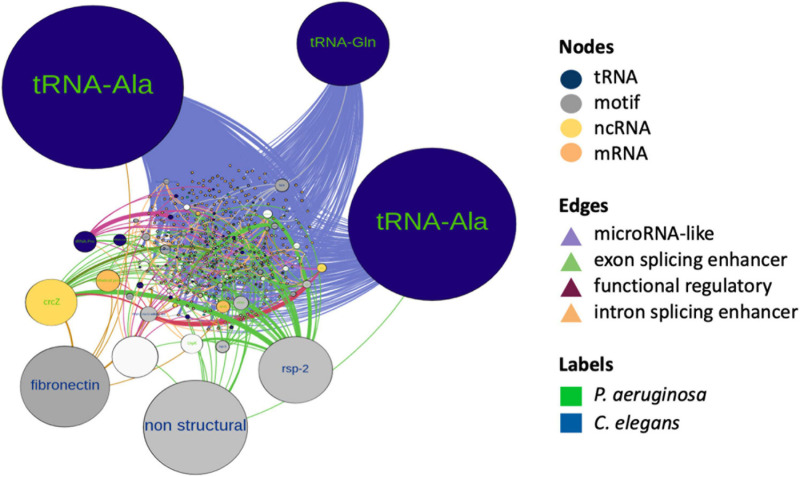
RNA interactome network of cross-kingdom predictions between bacterial non-coding sRNAs and host sRNAs and long non-coding RNAs and coding RNA candidate targets. Node colors represent biotypes and are differentiated by organisms. Nodes are scaled by size according to their betweenness centrality. The edges represent predicted interactions of this work and are colored according to the putative mechanism.

To gain insight on co-occurrence and co-regulation between the bacterial sRNAs and host targets, we integrated all the mechanisms of interspecies interaction proposed in this work and constructed a global RNA interaction network of bacteria and host putative interactors (Supplementary File 3). In order to classify the main interrelations, we use as input those *P. aeruginosa* RNAs that emerged as *induced*, *constitutive*, *microRNA-sized*, and *common TPs*, from the predictions of this work. Additionally, *C. elegans* transcripts that qualified as targets in any of the aforementioned predictions were used as nodes. Attributes considered for nodes were as follows: differential expression, organism type, biotype, and in bacteria, whether the sRNAs are *induced* or *constitutive* and *microRNA-sized* or not. The network consisted of 456 nodes and 1,569 edges, with an average weighted degree of 5.7 and 6.8, respectively, and a power law degree distribution. Network metrics are reported in Supplementary File 3. Nodes that correspond to *C. elegans* were 78.4, and 21.6% to *P. aeruginosa*. The most represented biotype in the network was host mRNA regulatory motifs and bacterial microRNA-sized sRNAs, followed by tRNAs and tRNA fragments of both species. We discriminate influential nodes based on the betweenness centrality and closeness centrality metrics (Figure 6). The biotypes with the higher betweenness centrality were tRNAs and tRNA-related motifs, from both species. Nodes with the higher betweenness centrality in bacteria were enriched in quorum sensing-related processes, such as *RhlE*, *RgsA*, and *CrcZ*. We also found an alternative splicing motif of fibronectin (EDA exon) as relevant. The modularity analysis renders four main modules. The cluster with the highest number of nodes corresponded to microRNAs’ predicted mechanism. The integration of RNA interactions in this network allowed us to uncover potentially relevant players of PIDF and pathogenesis in our paradigm, some of which can be further tested by mutant and phenotypic analysis.

### *In vivo* Phenotypic Analysis

**FIGURE 7:**
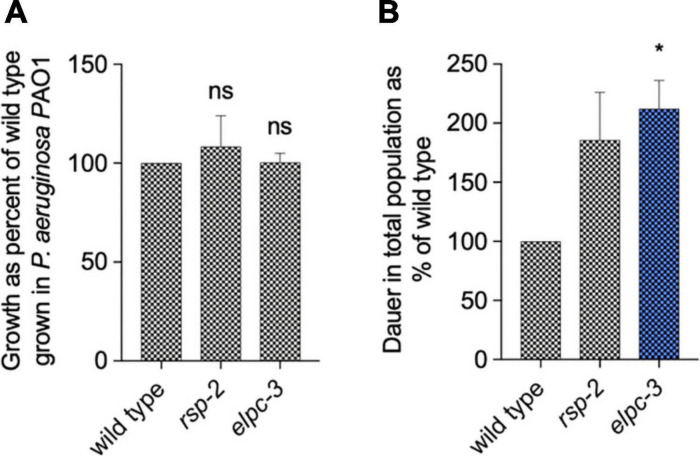
Growth **(A)** and diapause formation **(B)** of wild-type, *elpc-2*, and *rsp-2 C. elegans* mutants on *P. aeruginosa* PAO1 for two generations. *P < 0.05.

Through the integration of diverse *in silico* analysis, we found that the overrepresentation of tRNA motifs and RNA editing sites suggested that base modification in tRNAs could be implicated in interspecies interaction. Previous works have shown that tRNA base modifications determine *C. elegans* behavior (Fernandes De Abreu et al., 2020). We tested the possibility that modified tRNAs are good candidates for testing interspecies elicitation of PIDF. We asked whether worms that lack the ability to modify tRNAs show abnormal PIDF. We chose mutant nematodes defective of *elpc-2* and *elpc-3*, an elongation factor required for tRNA base modification, to study their potential role in PIDF. We first quantified the growth of *elpc-3* and *rsp-2* animals in *P. aeruginosa* PAO1 and compared it with wild-type animals. Figure 7A shows that growth of mutants is similar to the wild-type strain, indicating that the lack of *elpc-3* and *rsp-2* does not impair growth nor the wild-type innate response required for development under mild pathogens. We then quantified dauer formation after two generations feeding on *P. aeruginosa* PAO1. Interestingly, both mutants were able to form large numbers of dauers in pathogenic foods (Figure 7B), showing that neither is defective in PIDF. Furthermore, *elpc-3* animals formed significantly more dauers under pathogens, suggesting that tRNAs are halting the dauer defensive mechanism.

## Discussion

We investigated putative mechanisms by which RNAs from *P. aeruginosa* could target host RNAs and proteins to promote a heritable response and memory of the infection. We use the PIDF transgenerational paradigm of defense against pathogens where animals enter diapause when exposed to harmful bacteria. The analysis is based on RNA transcripts since sRNAs from *C. elegans* and bacteria have been described as necessary for PIDF (Gabaldón et al., 2020; Legüe et al., 2021). We approached this question by applying *in silico* tools that operationalize possible mechanistic frameworks of interaction between RNAs from two species. These analyses generate potential candidate triggers and targets of the interspecies communication to be tested *in vivo* for their role in specific life history traits. For example, here we show that effectors of tRNA modifications are relevant for wild-type PIDF.

### From Context-Specific RNA Expression to *in silico* Tools to Address RNA-Based Interspecies Communication

We selected the PIDF behavioral paradigm that results from the RNA interplay between *P. aeruginosa* and *C. elegans* for two generations. PIDF is triggered rapidly after animals are re-exposed to pathogens after having been withdrawn for two generations. We previously produced transcriptomic profiles of animals and their intestinal bacteria for two and six generations, respectively (Gabaldón et al., 2020; Legüe et al., 2021). We focus on bacterial sRNAs always upregulated in the intestines of the nematodes (induced sRNAs) compared to naïve conditions and contrast them with sRNAs constitutively expressed across generations. We complemented the conventional sRNA annotation with the information of the peaks of expression (TPs, Gabaldón et al., 2020), enabling the scrutiny of novel sRNAs and RNA fragments with potential biological relevance. The dual RNA-sequencing analysis allows us to study the simultaneous gene expression changes during the pathogen–host interaction.

The interaction analysis we perform is built on the following assumptions: First, RNA from both species is highly mobile, capable of being transferred between organisms by RNA transporters or membrane vesicles. These RNA species constitute the holo-transcriptome where RNAs expressed by either species are susceptible to interacting with each other. The RNA expression is context-dependent and tissue-specific. While we have taken context-specific transcriptomics data in highly synchronized populations, the extraction was done from whole animals. A form to solve this shortcoming would be to use data from single-cell-type transcriptomics in future investigations. Alternatively, individual interactions predicted by our analysis could be tested in specific tissues and under specific conditions. Second, we assume that RNAs induced under encounter with the *C. elegans* intestine are those most relevant for the interaction with the host because it acknowledges the connection of functionally relevant transcripts with PIDF. Third, we consider the pervasiveness of transcription (Leitão et al., 2020) and use a method that estimates as equally likely the expression of transcripts that have been previously annotated and those unannotated or expressed from intergenic regions.

The first mechanism evaluated was the interspecies post-transcriptional inhibition triggered by perfectly or incompletely paired sequences such as siRNA, miRNA-like, and acting sRNAs (asRNA). We also implemented approaches previously reported such as genomic alignment (Celluzzi and Masotti, 2016; Kaletsky et al., 2020). For siRNA exploration, we conducted the mapping of reads with Bowtie2, which is a validated and standard tool for global alignment. We chose this tool based on its use (Celluzzi and Masotti, 2016) even though the global alignment is not the best choice for dissimilar sequences such as those from different species. Therefore, we looked for paired transcripts with local alignment using BLAST (Camacho et al., 2009) and restricted it to expressed fragments, which allowed us to find less but more precise matches. It is relevant to highlight that the BLAST alignment does not constitute evidence for homology by itself but of sequence similarity. For selecting biologically relevant sequences, it is imperative to filter these results by *e*-value. We selected those with an *e*-value lower than 0.01, keeping in mind that small fragments have more chance of aligning randomly. This threshold is more restrictive than the one used by other authors (Kaletsky et al., 2020). Furthermore, even though interspecies matching sequences meet the statistical criteria of significance, they have a bit-score lower than 50, so we do not call them homologous despite their similarity. We additionally use the reciprocal best-hit analysis (Ward and Moreno-Hagelsieb, 2014), which consists in keeping the alignments in which both sequences are the best match of each other. This method was first used for finding coding sequences or protein orthologs but proved helpful in our analyses to keep the best matches between species.

The other post-transcriptional mechanism explored was the interactions between bacterial sRNA microRNA-sized with *C. elegans* mRNAs. In bacteria, *trans*-acting sRNAs (*trans*-asRNAs) share many characteristics with eukaryotic miRNAs (Layton et al., 2020) such as a limited base-pairing mechanism requiring only partial complementarity to their target sequence and a seed region of 7–12 nucleotides (Wachter et al., 2019). Eukaryotic miRNA function requires the RISC, which is absent in bacteria. However bacterial Hfq protein analogously presents and stabilizes sRNAs (De Lay et al., 2013; Diallo and Provost, 2020). *Trans*-asRNAs are typically 100–1,000 nucleotides, in contrast to microRNAs that are 21–25 nucleotides (Layton et al., 2020), but we know that shorter RNAs in bacteria have been largely overlooked despite the indications that they could be relevant players in interspecies communication (Zhao et al., 2017; Felden and Gilot, 2018; González Plaza, 2020; Layton et al., 2020). Interestingly, we found microRNA-sized fragments in the group of sRNAs responsive to interaction or *induced RNAs*, but not in the constitutive sRNA group. We found that these microRNA-sized transcripts mainly target intestinally expressed host mRNAs. This fact is concordant with our model in which only live bacteria colonizing the intestine can induce the PIDF response. These results allow us to speculate that microRNA-sized RNAs could be affecting locally the transcriptional state of the host at the site of the infection.

The evaluation of splicing regulatory motifs in bacteria–host interaction could appear counterintuitive, given that alternative splicing is not a relevant mechanism of gene expression control in bacteria. However, there is increasing evidence of drastic changes in host splicing regulation during infection (Liang et al., 2016; Kalam et al., 2018; Chauhan et al., 2019). We evaluated the presence of splicing regulatory motifs in bacterial sRNAs and found plenty of predicted enhancer and silencer intron and exon splicing regulators. These motifs were found in both induced and constitutive sRNAs with splicing silencer motifs slightly over-represented in the induced sRNAs. Much needs to be learned on how pathogens target splicing regulation in new scenarios to fully understand the relevance of these motifs in bacterial sRNAs. An open question is whether sRNAs from bacteria can bind to regulatory proteins in the host nematode as they do in bacteria. RNA-binding proteins (RBP) are involved in the processing, stability, and activity of sRNAs, providing precision in sRNA–mRNA base-pairing (Quendera et al., 2020). As newer tools are developed, it would be appropriate to test whether bacterial sRNAs that bind RBP from bacteria could also bind their orthologs in the nematode.

Finally, our analysis highlights tRNAs and tRNA-derived small RNAs (*tsRNAs*) as candidate mediators for interspecies interaction. The challenge of *elpc-3* mutants with *P. aeruginosa* pathogenic bacteria renders increased PIDF, suggesting that tRNA processing is necessary for the wild-type response to infection. tRNAs and tsRNAs have increasingly recognized roles in gene regulation under stress and in intergenerational inheritance (Chen et al., 2016). Indeed, some tsRNAs can be associated with Argonaute proteins and function as miRNAs (Shen et al., 2018). We found that tRNA-derived sRNAs were exclusively *induced sRNAs*, suggesting a role for tRNAs in our paradigm that may transcend their role in protein translation. Moreover, tRNAs decode essential mRNAs for protein synthesis, deliver amino acids to other places in the cell, and under stress conditions can be cleaved to generate signaling molecules or regulate gene expression (Raina and Ibba, 2014; Megel et al., 2015; Fields and Roy, 2018; Oberbauer and Schaefer, 2018; Barraud and Tisné, 2019; Nguyen et al., 2019).

### Relevance of RNA–RNA Communication in Behavior and Physiology

Bacterial RNAs are capable of triggering behavioral decisions in their nematode host (Kaletsky et al., 2020; Legüe et al., 2021). How do RNAs from the two species interact with each other in the context of a holobiont? Interspecies interactions and communication involve the bidirectional transport of regulatory molecules. At least two mechanisms are formally possible: sRNA movement through specific RNA transporters and the use of membrane vesicles for their delivery from the intestine to specific tissues. *C. elegans* expresses intestinal dsRNA transporters (SID-1, SID-2, and SID-5) needed for systemic and environmental RNAi (Winston et al., 2002, 2007; Hinas et al., 2012). Theoretically, these transporters could also internalize RNAs derived from colonizing bacteria (Legüe and Calixto, 2019). Bacterial cargo can be released through membrane vesicles of diverse nature. One well-documented example is through OMVs. These vesicles are known to transport a variety of cargoes including RNAs, proteins, and toxins, among other molecules, and play important roles both in pathogenesis (Ghosal et al., 2015; Koeppen et al., 2016; Zhang et al., 2020) and symbiosis (Moriano-Gutierrez et al., 2020). Additional vesicles that can carry RNA cargo are MV resulting from the explosive lysis of bacteria (Turnbull et al., 2016; Toyofuku et al., 2017).

In summary, this study offers a framework to analyze global transcriptomics in the context of survival behaviors against pathogenesis. The complexity of the interplay at the RNA level in interspecies communication underscores that behavioral adaptations are multistep strategies that require the integration of multiple effectors and targets.

## Data Availability Statement

The datasets analyzed during the current study are available in the NCBI repository under BioProject no. PRJNA659467 (https://www.ncbi.nlm.nih.gov/bioproject/PRJNA659467, Gabaldón et al., 2020) and BioProject no. PRJNA708299 (https://www.ncbi.nlm.nih.gov/bioproject/PRJNA708299, Legüe et al., 2021).

## Author Contributions

ML and AC made significant contributions in the conceptualization of the study, performed the methodology, wrote the original draft, and reviewed and edited the manuscript. AC acquired funding for the study. All authors conducted the investigation.

## Conflict of Interest

The authors declare that the research was conducted in the absence of any commercial or financial relationships that could be construed as a potential conflict of interest.

## Footnotes

1 http://www.bioinformatics.babraham.ac.uk/projects/fastqc

2 ftp://ftp.ncbi.nlm.nih.gov/genomes/all/

## Supplementary Material

Supplementary Figure 1 **(A,B)** Tracks in Genome Browser of read location and coverage. gff files loaded onto Wormbase Genome Browser to show the genomic context where the reads are located.

Supplementary Figure 2 Venn diagram showing shared putative *C. elegans* targets of sRNAs microRNA-sized that are *induced* and expressed in *C. elegans* intestines from *P. aeruginosa*.

Supplementary Table 1 *Caenorhabditis elegans* genomic annotations mapped by naïve *P. aeruginosa* PAO1 reads. List of *C. elegans* genes mapped by *P. aeruginosa* reads expressed in host-naïve conditions.

Supplementary Table 2 Host-induced sRNAs from *P. aeruginosa* PAO1. Bacterial sRNAs upregulated in the F1, F2, F5, and F6 *C. elegans* generations compared to naïve bacteria.

Supplementary Table 3 Constitutively expressed sRNAs from *P. aeruginosa* PAO1. Genes whose expression does not change in intestinal conditions compared to naïve bacteria.

Supplementary Table 4 All transcripts expressed in intestinal conditions with their lengths. Lists of all genes expressed in any of the F1, F2, F5, and F6 generations of *C. elegans* and further subdivisions as induced and microRNA-sized.

Supplementary Table 5 Transcripts constitutively expressed with their lengths.

Supplementary Table 6 *Caenorhabditis elegans* mRNA putative targets of microRNA-sized-induced sRNAs from *P. aeruginosa* PAO1.

Supplementary Table 7 List of *C. elegans* mRNA targets of *P. aeruginosa* miRNA-like sRNAs expressed intestinally in *C. elegans*.

Supplementary File 1 All putative interactions of microRNA-sized host-induced sRNAs with *C. elegans* mRNAs downregulated during interaction with *P. aeruginosa* PAO1.

Supplementary File 2 List of regulatory motifs predicted to be targeted by induced and constitutive sRNAs by RegRNA 2.0.

Supplementary File 3 Data for construction of the RNA interactome network.
