# Supplementary figures and images for: Interspecies RNA Interactome of Pathogen and Host in a Heritable Defensive Strategy
Source: Front Microbiol. 2021 Jul 21;12:649858. doi: 10.3389/fmicb.2021.649858 (PMC8334366; doi:10.3389/fmicb.2021.649858)

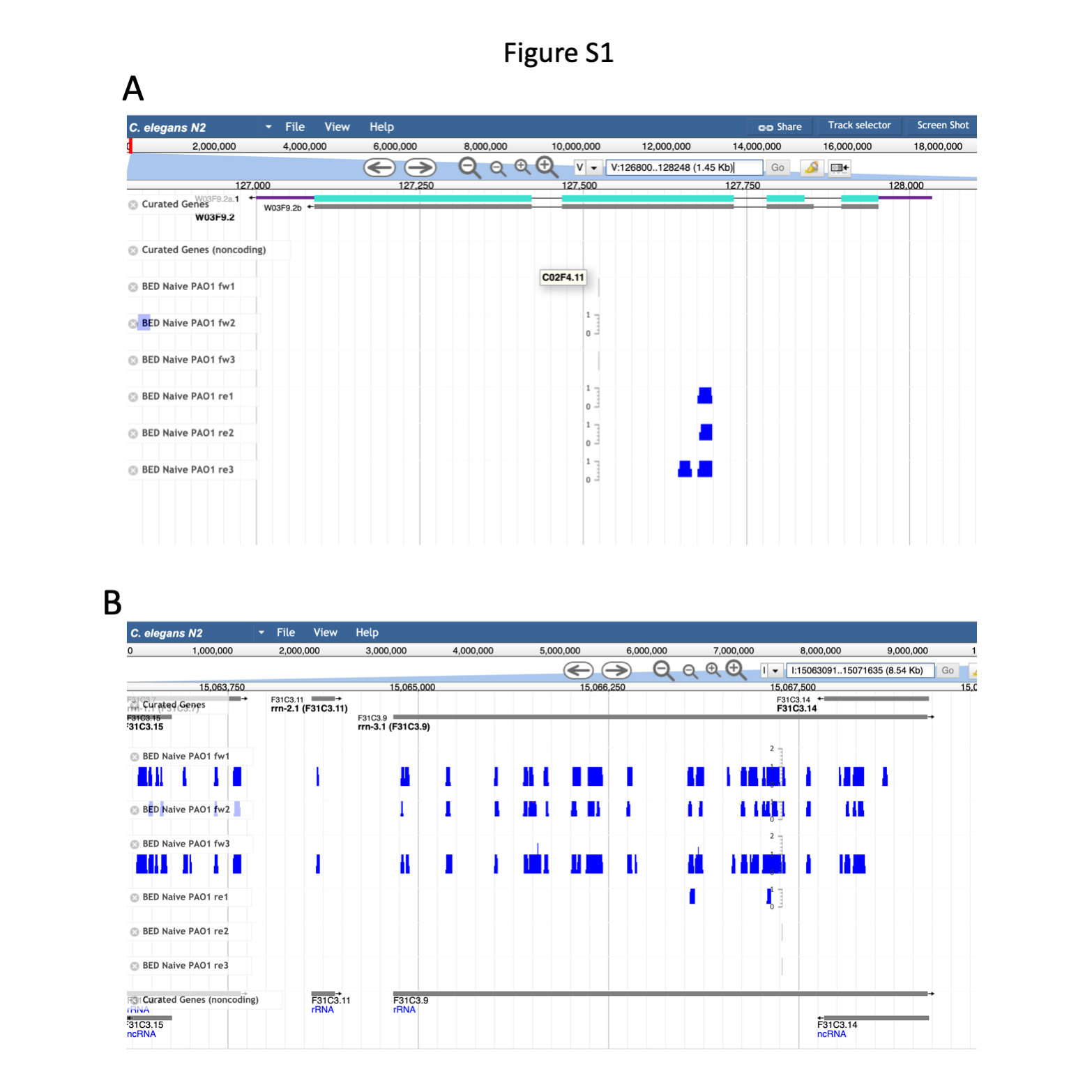

Supplement: Supplementary Figure 1 — (A,B) Tracks in Genome Browser of read location and coverage. gff files loaded onto Wormbase Genome Browser to show the genomic context where the reads are located. [file Image_1.TIFF]

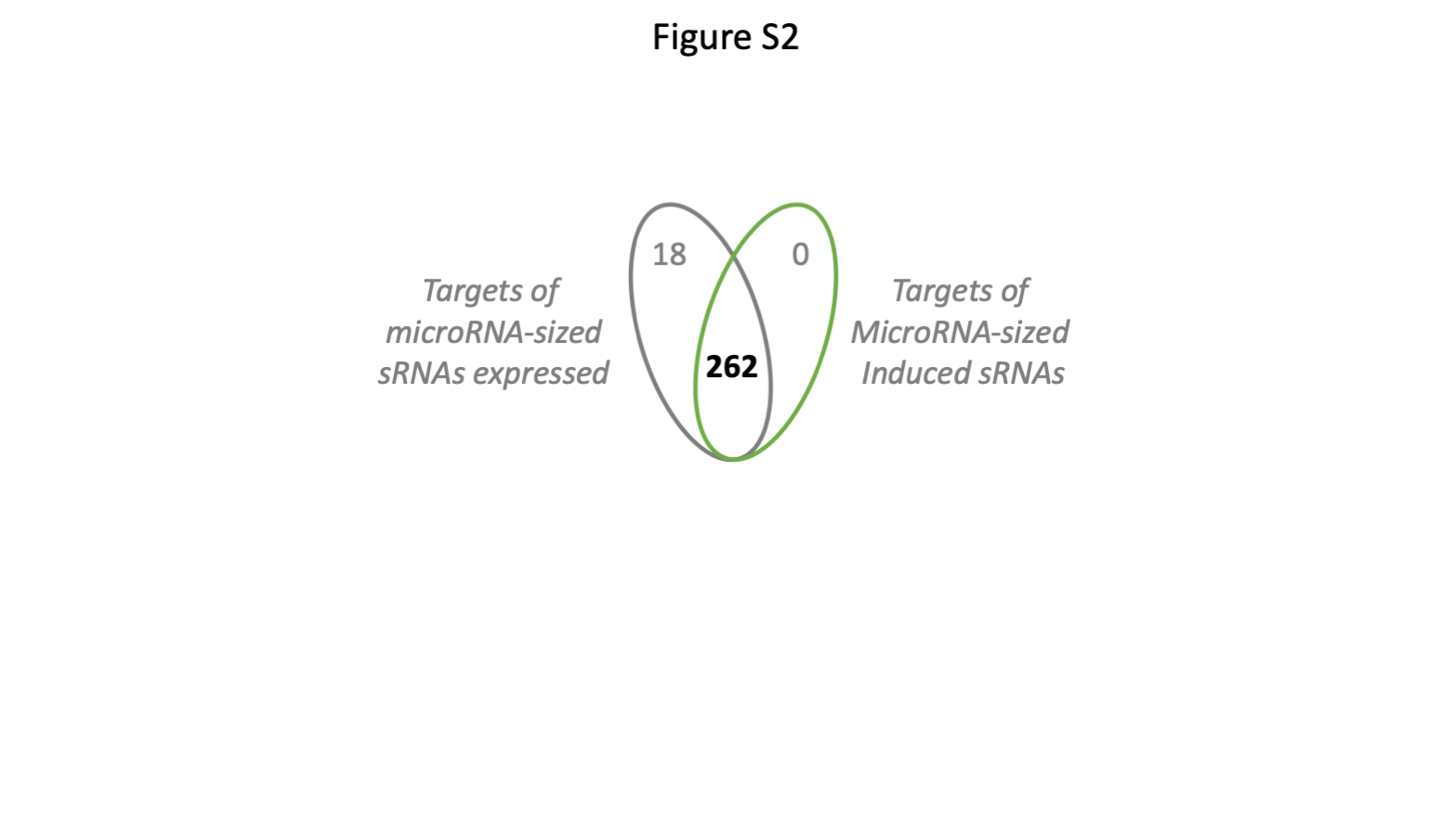

Supplement: Supplementary Figure 2 — Venn diagram showing shared putative C. elegans targets of sRNAs microRNA-sized that are induced and expressed in C. elegans intestines from P. aeruginosa. [file Image_2.TIFF]
